# Grand Challenges in Global Health: Engaging Civil Society Organizations in Biomedical Research in Developing Countries

**DOI:** 10.1371/journal.pmed.0040272

**Published:** 2007-09-11

**Authors:** Anant Bhan, Jerome A Singh, Ross E. G Upshur, Peter A Singer, Abdallah S Daar

## Abstract

The authors discuss the different types of civil society organizations, their role in biomedical research, and the advantages and challenges of working with them.

Civil society organizations (CSOs) are nonprofit organizations that aim to further the interests of the communities they serve. Driven to protect and empower the vulnerable, CSOs work in areas such as community development, service provision, advocacy, activism, and research.

Sanders and colleagues argue that CSOs are at the forefront of supporting innovations aimed at tackling today's global public health challenges and that working with them plays a crucial role in making research relevant to communities [1]. While several publications have addressed the role of CSOs in social science research in health [2–4], their role in biomedical research has not been widely discussed.

The recent termination of tenofovir-based HIV prevention trials in Cambodia, Cameroon, and Nigeria, primarily as a result of pressure from CSOs, underscores the significant impact CSOs can have on biomedical research [5]. In Cambodia, in particular, intense pressure from CSOs influenced the Prime Minister's decision to suspend a trial of tenofovir (an antiretroviral medication used to treat HIV) among commercial sex workers [5]. Similar outcomes have occurred in other countries hosting tenofovir prevention studies, and have complicated potentially promising research on this drug [6,7]. The termination of these trials has reinforced the importance of engaging with communities and CSOs, and being sensitive to their respective needs and perceptions.

In October 2005 the Bill & Melinda Gates Foundation announced an approximately US$450 million sponsorship of 44 innovative projects under the auspices of its Grand Challenges in Global Health (GCGH) initiative. As described in the opening article in this series, we serve as an advisory service on ethical, social, and cultural (ESC) issues for these projects [8]. We are exploring a range of ESC issues identified by the GCGH investigators and independently by developing world key informants in a study published as the second paper in this series [9]. The investigators and key informants placed particular emphasis on the importance of engaging with CSOs in research, and therefore we prepared a conceptual paper on this topic, which we distributed as a working paper to GCGH investigators and program staff at the 2nd Annual GCGH Meeting held in Washington, D. C. in October 2006.

This work represents the final version of our analysis. Our article aims to delineate different types of CSOs, their role in biomedical research, and the advantages and challenges of working with them. We stress that despite the challenges, it is in the best interests of science and researchers working in the developing world to identify and engage with CSOs. We also argue that there is a need for empirical research on best practice models of CSO–researcher engagements and for evaluation of these models.

## CSOs in the Developing World

CSOs are common in the developing world. In Bangladesh, for example, one study found at least one CSO in over 90% of villages surveyed in 2000 [10]. The Bangladesh Rural Advancement Committee (http://www.brac.net/), which is involved in health services provision throughout the country, has in recent years founded a university, including a public health school to train public health researchers and practitioners. CSOs can be divided into five types:

### Nongovernmental organizations

Nongovernmental organizations (NGOs) work outside the direct control of governments. They can be as large as Médecins Sans Frontières or Greenpeace, or small and located in a single developing country village. The World Bank describes NGOs as “private organizations that pursue activities to relieve suffering, promote the interests of the poor, protect the environment, provide basic social services, or undertake community development” [11]. NGO activities can be local, national, or international [12].

### Community-based organizations

Community-based organizations draw their membership from the communities they serve. While most have leaders, decisions are typically collective in nature. Self-help groups of women in a village involved in microfinance initiatives are an example of such a body.

### Faith-based organizations

Faith-based organizations draw the purpose of their work from a particular faith or religious belief and may work through local centers of faith, such as churches, mosques, or temples. Examples include the Salvation Army, the YMCA, and the Ramakrishna Mission. Collaborating with such bodies would be especially useful in countries that are home to distinct religious groups. A recent United Nations study acknowledged the important role faith-based organizations play in responding to HIV/AIDS, especially in sub-Saharan Africa, and called for “greater collaboration between them and public health agencies” [13].

### Voluntary health organizations

Voluntary health organizations are patient advocacy organizations, often focused on a single disease or syndrome, that promote research and participation in trials, treatment access, and aid for those affected by disease. They regularly discuss health issues with policy makers and the public. Examples include the Global Network for People Living with HIV/AIDS, the Eye Bank Association of India, and Diabetes South Africa.

### Networks

Networks are groups comprising various organizations and individuals that converge around common issues. Examples include the global People's Health Movement and the Right to Food Campaign in India.

Gilson identifies four main health sector functions for CSOs: service provision, social welfare activities, support activities, and research and advocacy [14].

In relation to research, Jacob and Price suggest that investigators, especially those in externally funded projects with short time frames, should identify and work with local CSOs in engaging with communities [15]. Fruttero and Gauri point out that CSOs can “contribute to all different stages of the research cycle, namely in advocacy, priority setting, capacity building, resource mobilization, sharing and utilization of research findings, networking, and ethical assessment of research being carried out” [10].

Sanders et al. have identified three main ways in which the participation of CSOs in research, as users or generators of research, can be increased. These are “influencing commissioning and priority-setting [of the research, to be relevant to local health needs]; becoming involved in the [peer] review process and in conducting research; and through formal partnerships between communities and universities that link CSOs with academic researchers” [1]. Zafar Ullah et al. examined collaborations between the government and CSOs in Bangladesh in tuberculosis control, and found that such partnerships can lead to better access and quality of services provided [16]. Box 1 gives specific examples of how CSOs can play a role in scientific and medical research.

**Box 1. Examples of CSOs Playing a Role in Research**

- CSOs can conduct research and facilitate technology development. An example is the Program for Appropriate Technology and Health (http://www.path.org/), which conducts frontline research in the developing world to develop health solutions of relevance to that region, independently or in partnership with other researchers.
- CSOs can have a positive impact on research agendas by exerting pressure on governments and researchers. For example, the Treatment Action Campaign in South Africa pressured the South African government into halting its ethically questionable research pilot program on nevirapine in pregnant mothers with HIV at designated research sites, convincing them to provide it to pregnant mothers with HIV nationwide.
- CSOs can disrupt scientific endeavors and arguably jeopardize science if they feel the scientific premise is not valid or harmful to the communities they serve. For example, activists have campaigned against genetically modified organisms, and Act UP Paris campaigned to halt the tenofovir trials in Cambodia and Cameroon [5].
- CSOs can play important advocacy roles. For example, voluntary health organizations sometimes focus on a specific disease, such as diabetes, and undertake advocacy work with policy makers and funders to increase the level of funding for researching those diseases.

There is a widespread perception that many CSOs have an anti-science agenda. This perception probably arose from the fact that the issues involving CSOs that attract media attention are contentious ones, such as environmental degradation (e.g., involving Greenpeace), genetically modified organisms (e.g., involving GM Watch and Gene Campaign), and intellectual property rights, particularly in reference to essential drugs (e.g., involving the Treatment Action Campaign in South Africa). Such a perception is misleading, as some CSOs play a pivotal role in promoting science. For example, Bharat Gyan Vigyan Samithi (BGVS or the Indian Organization for Learning and Science) works in many parts of India to make science education widely accessible. CSOs are also represented on the various advisory boards of the International AIDS Vaccine Initiative.

Widdus identifies four challenges facing organizations claiming to be working in the public interest: “representation of intended beneficiaries, funders and other stakeholders; conflicts of interest, which include biases arising from any person's organizational affiliation or strongly held convictions; accountability; and transparency” [17]. Widdus also cautions that while engaging with CSOs is useful, local elected governments should not be neglected.

There have been instances where well-endowed foreign CSOs have provided financial incentives to communities to attend their meetings, and this has put poorer, local CSOs at a disadvantage in getting the attention of the local communities [18]. Researchers must be careful to ensure that the missions of their partner CSOs align with the needs of the community rather than the donors. There are also instances where donors promise one thing but do something else on the ground [19]; this may result in lack of credibility on the part of CSOs and any researchers associated with them. Furthermore, researchers should be aware of potential conflicts of interest when CSOs are motivated primarily by a need to sustain donor interest in their work rather than responding to genuine needs of the communities they aim to serve.

Researchers should strive to work with accountable, transparent CSOs. A November 2006 article in the Washington Post noted how pygmy populations in central Africa felt exploited by the various CSOs collecting funds in their name. “So many local NGOs have come to visit and promised to build houses,” said a Pygmy chief quoted in the article. “But so far, nothing.” [20]. Working with CSOs in nations where governments restrict civil society also entails risks for researchers [21]. Collaborating with such CSOs may incur unwanted government harassment and scrutiny.

While community engagement in developing world research has been extensively discussed [22], the role of CSOs in increasing community engagement in biomedical research and enhancing the ultimate adoption of the resulting health policies and technologies has not been adequately explored.

## The Role of CSOs in Biomedical Research

We have devised the following preliminary taxonomy of six roles played by CSOs with an emphasis on community engagement in biomedical research.

### Community interface

CSOs can play a valuable interfacing role between researchers and communities on issues such as informed consent, negotiating ancillary care during trials and posttrial care/benefits, establishing community advisory boards, and illuminating sociocultural beliefs and practices. CSO members often hail from the communities in which they work and are generally trusted by community members. CSOs have intimate knowledge of the health needs of communities, and the hierarchical nature of relationships within them. They can therefore sensitize researchers to the community's particular sociocultural practices. CSOs often take on the role of cultural interpreters to explain the nature of the community to researchers and the nature of the proposed research to the community in a culturally appropriate manner.

### Access point

CSOs can help researchers access vulnerable or stigmatized communities, such as refugee populations or sexual minorities, who may be largely invisible and inaccessible to an external researcher unfamiliar with local customs, traditions, or power structures. The fact that CSOs are nongovernmental and distinguishable from governmental power structures also makes them more credible and trusted in communities that have been historically discriminated against by the state. The International Committee of the Red Cross, Médecins Sans Frontières, and Amnesty International are examples of CSOs that typically have access to vulnerable communities who might be unwilling to trust or access traditional government structures

### Researcher

Many CSOs are primarily research-focused and contain experienced biomedical and/or social scientists, representing a largely untapped resource for outside researchers. Such individuals could serve as collaborators on investigator-driven research in areas such as epidemiology, social sciences, product development, knowledge translation, health services, and policy. Examples of CSOs in India that are staffed by biomedical and social scientists, and that have done innovative research of relevance to public health in developing countries, include SEARCH (http://www.searchgadchiroli.org/) with its work on community health workers and maternal and neonatal health, and SANGATH (http://www.sangath.com/sangath/), which focuses on mental, reproductive, and sexual health.

### Touchstone

CSOs may be a valuable check to make sure that the research being planned and implemented is in the best interests of the local populations and communities and respects their views and rights

### Advocate

Given their experience in working with local policy makers and politicians, CSOs can play a valuable advocacy role. If research findings indicate that an intervention is efficacious and sustainable in a particular setting, CSOs can pressure local health authorities to make the intervention available in the public health sector in a timely manner and at an affordable rate.

### Distribution channel

In some cases, as in the example of the Bangladesh Rural Advancement Committee, CSOs can deliver effective interventions themselves.

## Next Steps

The full benefit of working with CSOs in biomedical research in the developing world is largely untapped, and it needs to be better understood. Where appropriate, researchers working in the developing world, including the GCGH investigators, should take advantage of working with CSOs. Engaging with CSOs has several benefits for researchers, especially in approaching and working with communities, and for post-research adoption of innovative findings and products. CSOs could play a key role in fostering understanding of how communities currently access technology and how they could do so in the future.

Despite the potential benefits of working with CSOs for community engagement in research, there are few empirical studies on best practices for collaboration with CSOs, or on the steps that research-based CSOs have taken in the process of community engagement. In the third paper in this series, we discussed how our project would examine community engagement in research through case studies [22]. In several of these cases we will be focusing on the role of CSOs. Empirical research and evaluation of best practice models for partnering with CSOs in biomedical research would be in the best interest of science and research in the developing world. 3

## Abbreviations

CSO: civil society organization
ESC: ethical, social, and cultural
GCGH: Grand Challenges in Global Health
NGO: nongovernmental organization
